# Here’s to You Little One

**DOI:** 10.4269/ajtmh.19-0234

**Published:** 2019-08

**Authors:** Raffaele Macri

**Affiliations:** Ross University School of Medicine, Bridgetown, Barbados

It was a hot summer afternoon in a small village along the southeastern coast of Madagascar, where I served as a Peace Corps volunteer. The grandmother of a 3-week-old baby boy knocked on my door. I knew who she was, as she was a prominent member of the community. “Rafahely can you come over to my house and see Etienne? He’s sick.” Tahina told me Etienne was having a lot of diarrhea and it had not improved. I lived next door to the health clinic, where the doctor was out of town for a few days attending a regional meeting and getting supplies. Tahina lived on the other side of the clinic. Both of us lived within a stone’s throw of the small four-room brick-and-mortar building, with no electricity or running water. We had access to medical care that Etienne needed, but receiving that care is another story.

The African island nation of Madagascar has a population of 25.5 million people, with 77.6% of the population making less than $2 per day.^1^ Although the country has many resources at its fingertips, including being one of the top exporters of vanilla beans, it is riddled with corruption. It is the 8th poorest nation in the world.^2^

I went to Tahina’s house and saw Etienne being held by her granddaughter, Lalasoa. His cries and angst were filling the room. I had experience being a medical assistant and I was trained to recognize the various danger signs and symptoms of medical conditions such as dehydration, marasmus, kwashiorkor, and malaria. I learned about it, but when the books turn into reality, that knowledge manifests as a need to assist in any way possible. I saw Etienne crying, with a sunken fontanelle and loose thin-leathery skin. I asked Lalasoa if I could hold him and it only took a few minutes of him being in my arms before he had a bout of profuse watery diarrhea.

I immediately remembered the lesson on oral rehydration salts (ORS), a simple at-home treatment for diarrhea and electrolyte replenishment—eight tablespoons of sugar and one tablespoon of salt in 1 L of clean water. “Valo sy iray”−“8 and one” as many of the locals had come to learn of the at-home treatment ingredients.

After cradling Etienne, hearing his cries, and observing his face and frail skin, I knew without treatment he would not last long.

In talking with Tahina, I learned that her daughter, the mother of Etienne, died during childbirth, as many women do in small developing villages. Childbirth complications include hemorrhage, in which the mother’s vessels fail to constrict, leading to never-ending blood loss; eclampsia, where ever-worsening high blood pressure leads to multiple organ failure; or sepsis, where infection resistant to treatment ravages the body. In most cases, the cause of death is unknown because of lack of resources.

Because of the death of his mother, Etienne could not be breastfed, but rather was formula fed. The formula was free from a mother-and-child health clinic 18 km north. Tahina could not afford the $1.25 fare to take the 30-minute bus ride, so she frequently walked. When she walked, she left in the early morning and arrived in the early afternoon, “It takes me 4–6 hours to walk there, and I don’t have the energy.” She would stay with a friend or family member in that town to rest for the walk home the following day.

For the formula to be effective, it needs to be mixed with a precise amount of “clean” water. Where are they getting this water from? In the Madagascar countryside, people obtain their water from many sources, including pumps, wells, lakes, or water runoff. Tahina’s main source of water was the lake behind her home, which was definitely contaminated. As latrines or outhouses were scarce, most people practiced open defecation.

Although she had the formula and knew how to mix it, was she feeding him frequently enough? Newborns should be fed 2–4 ounces of formula every 2 hours.

After talking with her and observing Etienne, I walked her back to my house and demonstrated how to make ORS. “Yes, I already know how to make this,” she said. “But I don’t have money for the salt and sugar.” I gave her a 1-L water bottle full of the solution to take home with her. I drew a line about half way on the bottle with a permanent marker to indicate how much Etienne should consume by nighttime. The entire liter of ORS should be consumed in 24 hours. She nodded in understanding and, with a large smile on her face, took the bottle.

There are many reasons in low-income countries why people don’t seek treatment. I wondered why she did not seek treatment from the health clinic because she clearly had access. Although the doctor was away, I sensed there were other reasons. Later, talking to her, I realized that she felt uncomfortable receiving care because of the corruption in the village and her lack of money to pay for care.

My eagerness to meet up with Tahina for a progress report on Etienne the next day was immediately met with despair. He had only consumed one quarter of the ORS solution, half of what was necessary for him to recover. In addition, his diarrhea had progressed, which is a definite sign of a poor prognosis.

Etienne passed away the next day. The following day was his funeral. I visited the house where he was wrapped in a silken white cloth in the middle of a bed. A mosquito net was draped around the bed posts to give him privacy.

In my medical career 3 years later, hoping my brain can act like a sponge as I sleep on my study aid books, this story still occupies space. I still think about that entire interaction with Tahina and Etienne. It was such a terrible situation, yet completely preventable.

During my pediatric rotation, administering vaccinations to children of all ages, one of them being rotavirus, it dawned on me! Perhaps, Etienne was suffering from a diarrheal infection from rotavirus, one of the leading causes of death worldwide in children aged less than 5 years. The rotavirus vaccination is required as two doses at the ages of 2 and 4 months, or three doses at the ages of 2, 4, and 6 months in the United States. It is administered orally, a much more pleasant administration method than the classic injection.

What if the rotavirus vaccine were delivered and accessible to children across Madagascar? Would Etienne have survived? Would Tahina, who I had grown to love, have had to suffer through the perils of taking care of her grandson in such a traumatic and deteriorating way? The vaccination requires refrigeration, a factor that many clinics and hospitals in the United States have no trouble addressing. Could this small village, which does not have the infrastructure or the electrical or solar energy yet to power a refrigerator, ever have access to such a vaccination? In a country like Madagascar, this could be lifesaving.

I was ecstatic to return to my old village 6 years later as an American Society of Tropical Medicine and Hygiene Ben Kean Travel Fellowship recipient and learn from patients in that very clinic where I volunteered. I witnessed the effects that international aid has on the local communities. The same four-room clinic now has electricity in every room powered by solar panels; a refrigerator for vaccines, rotavirus being one of them; and even a running water system from a nearby water tank collected from rainfall, an essential component during the rainy cyclone season. The clinic now has enough staff to be accessible at any hour. All of these perhaps could have saved Etienne.

When I think of this story and Etienne, I think about the future of health care. Health care is a basic human right, not a privilege. The public needs to be educated on the right of health care. Perhaps, more behavior change education needs to take place, in order for the public to feel comfortable receiving care. Once we provide resources and education, I believe, we will slowly start to see a change in health-care disparities. Accessibility does not just mean proximity. Over the course of 6 years, those resources became available to that small village. Perhaps, within those 6 years of waiting, more children perished. Tahina lived right next door to the clinic; she was close, but yet something was prohibiting her from going.

I write this story during a family medicine rotation, in between patients, as I wrap up my third year of medical school. I write this story in hopes of educating that younger version of me and in hopes of emphasizing that education is one of the many important factors that we have to improve the health of the underserved.
